# Reading Skill Profiles in School-Aged Italian-Speaking Children: A Latent Profile Analysis Investigation into the Interplay of Decoding, Comprehension and Attentional Control

**DOI:** 10.3390/brainsci14040390

**Published:** 2024-04-17

**Authors:** Angela Pasqualotto, Noemi Mazzoni, Francesco Benso, Carlo Chiorri

**Affiliations:** 1 Department of Psychology and Cognitive Science, University of Trento, 38068 Rovereto, TN, Italy; noemi.mazzoni@unitn.it (N.M.); francesco.benso@unitn.it (F.B.); 2 Department of Education and Learning, University of Applied Sciences and Arts of Southern Switzerland, 6600 Locarno, Switzerland; 3 Faculty of Psychology and Educational Sciences, University of Geneva, 1205 Geneva, Switzerland; 4 Department of Educational Sciences, University of Genoa, 16126 Genoa, GE, Italy

**Keywords:** reading, decoding, comprehension, attentional control, working memory

## Abstract

Our study examined the complex relationships among reading performance (decoding, comprehension) and language, visuo-spatial, and attentional control abilities in 115 Italian-speaking children. Latent profile analysis was used to identify distinct clusters of participants showcasing quantitative differences in decoding skills, including word, pseudo-word, text reading speed and accuracy. Then, we used this classification to investigate group differences in a variety of linguistic, working memory, and visuo-spatial tasks, as well as in reading comprehension skills, by means of multivariate and univariate tests. Our results reveal significant links between reading proficiency and several key factors: language skills, visuo-spatial abilities, and attentional control. These findings illuminate the nuanced impact of domain-general processes that govern a series of linguistic and visuo-perceptive subcomponents during reading tasks. Additionally, using dominance analysis, predictors of written text comprehension were identified. Our findings suggest that effective reading comprehension relies on a synergistic interplay of adequate reading speed, attentional control, working memory, and verbal fluency, accounting for 23% of the explained variance. This study highlights the multifaceted nature of reading proficiency and suggests that a broader perspective is necessary to fully understand reading development and support its improvement.

## 1. Introduction

Reading refers to an active cognitive process that involves decoding symbols to construct meanings from what has been read. Specifically, reading fluency includes decoding and comprehension abilities as well as prosody. Indeed, the ultimate goal of reading is not simply to decode rapidly and accurately but to fully understand what has been read [1]. Learning to read fluently occurs only when the decoding and comprehension skills enable individuals to read fast, accurately, and with prosody. As Burns and Kid [2] pointed out, “a focus on any one aspect of learning to read should not be at the expense of an emphasis on other aspects” (p. 1).

Despite the fact that for most readers, “learning to read” represents a simple and effortless process, many children have extreme difficulty in the acquisition and automatization of even basic reading skills (e.g., phonics). In this regard, it has been estimated that between 5% and 10% of school-aged children can be classified as “poor readers” and/or “poor comprehenders” [3,4].

A lack of good reading skills can negatively affect not only academic performance but also self-esteem, sense of self-efficacy, and, more generally, social adaptation (e.g., [5,6]). Therefore, addressing poor performance in reading represents a fundamental challenge for the entire society.

The starting point should be to understand the early development of reading skills in poor readers/comprehenders and which factors influence this development. Previous studies [7,8] have recognized the critical role played by some domain-specific skills (e.g., phonological awareness, orthographic knowledge, morphological awareness, and rapid automatized naming).

When exploring the visuo-spatial dimension, research by Livingstone et al. [9] and Best and Demb [10] has highlighted the significance of the magnocellular system, albeit with lesser emphasis on auditory modalities. Additionally, Geiger and Lettvin [11] identified “asymmetric crowding”—the difficulty dyslexics encounter in grouping words and letters, particularly on the visual field’s right side—as a factor in reading challenges. Furthermore, Fawcett et al. [12] described a poorer performance in cerebellar-related tasks among dyslexic individuals compared to children without reading difficulties matched for age and IQ. Bakker’s balance model [13] proposed a multi-component framework (encompassing linguistic, visual–perceptual, and mixed aspects) to account for the diverse challenges faced by dyslexics, including Italian speakers.

Moreover, recent research has emphasized the crucial role played by domain-general cognitive functions, particularly executive components, in determining the automatization of reading [14,15,16,17,18]. Indeed, some research indicates that these domain-general processes may be considered an even stronger predictor of early academic achievement than psychometric intelligence [19,20].

Hence, cognitive abilities, either domain-specific or general, form the bedrock upon which reading skills are built, preceding their acquisition and automatization [21,22]. In the subsequent sections, we will review empirical evidence concerning the relationship between executive components, such as attentional control, and reading.

### 1.1. Attentional Control: A Fundamental Pillar of Reading Skills’ Development

Considerable research has highlighted the significant role of cognitive skills in shaping the development of key reading abilities such as decoding and comprehension (for a recent meta-analytic review, refer to [23]). This body of work suggests potential variations in the associations between distinct cognitive skills and different facets of reading proficiency. However, the contributions of various executive processes in determining reading proficiency remain underexplored. It stands to reason that not all executive functions contribute uniformly or equivalently to diverse reading skills. Yet, prior studies have predominantly examined the influence of individual executive skills in isolation. Notably, this fractionated view of executive components could be misleading because most of the tasks that measure specific executive components recruit and involve not only the assessed function of interest but also other non-targeted cognitive processes as well as measurement errors (i.e., the “task impurity problem”, [24,25,26,27]).

For these reasons, in the present study, we chose to investigate the role of executive processes broadly. To this aim, as a theoretical background, we adopted the broader concept of “attentional control” (also called “executive control” or “executive attention”), namely a limited-capacity network that is specialized for detecting and resolving conflicts between competing processes [28,29,30,31]. Attentional control has also been conceptualized with respect to working memory capacity (WMC; [32,33]). As Engle stated (Engle, 2018, p. 191), WMC reflects “differences in ability to control endogenous attention—the ability to maintain attention on critical tasks and to avoid having attention captured by either internally generated thoughts […] or externally generated events […] that lead to thoughts that compete with performance on the task”. For instance, the perspective on individual differences in WMC through the lens of attentional control aligns well with the innovative theory of intelligence, known as “process overlap theory” [34]. It has been argued that domain-general executive attention processes act as a central bottleneck for task performance and serve as a constraint on the development of domain-specific cognitive abilities [35,36]. It is important to clarify that WMC has occasionally been mistakenly associated with “updating” [37] and simplistically portrayed as one single component of executive function [38]. Morra et al. [39] highlighted these theoretical misunderstandings, pointing out that adhering to the componential theory of working memory introduces logical inconsistency. Indeed, claiming that working memory serves as an executive function creates a circular argument, as working memory encompasses a central executive, which in turn includes executive functions. For a comprehensive overview of the contribution of information maintenance and disengagement to higher-order cognition, we refer the reader to Shipstead et al. [40].

In sum, attentional control could be considered a common thread linking performance in many complex cognitive tasks, particularly those requiring active goal maintenance and conflict resolution, such as decoding and comprehension of written material.

### 1.2. The Relationship between Decoding and Attentional Control

Decoding [41,42] refers to the process of reading by transforming graphemes into spoken units (e.g., phonemes, syllables) and then blending the units into pronunciations (e.g., spoken word or non-word). Extensive research has shown the importance of language processes (e.g., phonological awareness and rapid naming) in determining the speed and accuracy of decoding [43,44,45]. Besides language skills, attentional control plays a pivotal role in determining reading proficiency, as it allows the reader to flexibly allocate the focus of attention on what is relevant and ignore distractors [28,33,46]. Additionally, it is important to block out nearby phonological or orthographic distractions. This has been linked to decoding in a number of training, cross-sectional, and longitudinal studies (e.g., [47,48,49]). For example, the adequate sequential processing of the graphemes, comprised in each word by a scanning spotlight of attention, appears to crucially influence reading fluency [47,50,51]. Similarly, to efficiently decode a written page with a variable level of crowding, the focus of attention needs to be properly distributed on-page [52,53,54].

Attentional control also allows the flexible switching between different types of information (e.g., orthographic, phonological, and morphological) to be extracted from the same string of letters [55,56]. In this regard, it has been suggested that it allows the reader to flexibly manage orthographic, phonological, and morphological representations during lexical retrieval processes; it may be particularly relevant for languages with opaque orthography [55,57], and its association with decoding might diminish with age [58]. Moreover, individuals need to suppress irrelevant incorrect representations of graphemes or syllables during decoding, particularly when encountering homophones (words with identical pronunciation but distinct meanings and spellings) and homographs (words with the same spelling but often differing in pronunciation and meaning; [59,60,61]. However, there is no complete agreement on the direct role of inhibitory mechanisms (see, for example, Mac Leod et al. [62]). It is important to consider that attentional control encompasses inhibition but is not limited to it.

As we mentioned above, attentional control has been conceptualized with respect to WMC [32,33]. In this regard, evidence exists about reading proficiency being closely tied to working memory capacity, which supports readers in graphemes-to-phonemes mapping, facilitating the retention of phonological and morphological representations during decoding [63,64,65] (however, see [66,67] for controversial findings). In particular, studies have shown a link between verbal working memory and decoding skills [61,68,69] as well as the visual domain [66,70].

### 1.3. The Relationship between Comprehension and Attentional Control

A considerable proportion of the literature has addressed reading comprehension as one of the most complex cognitive activities that humans are able to perform [71] and has stressed its importance for the full development of the individual [1]. The construction–integration (CI) model [72] is one of the most often used theoretical models [14,73] and defines comprehension as the construction of a mental representation of what the text is about. According to this model, reading comprehension encompasses two steps: construction and integration. During the first step, construction, mental representations of the information contained in the text and background knowledge are activated by different sources of input: the current text, the sentence, the text that has been read before, and background knowledge. During the second step, integration, the activated information is connected into a network of concepts, and only the nodes that are strongly linked to each other within the network are maintained in the final version of the text’s representation. At the end of the reading process, the associative network of information, which has been fine-tuned, represents the mental representation of what the text is about. The selection of the proper representations, on the one hand, and ignoring conflicting or inappropriate information nodes, on the other hand, require the intervention of executive control [72].

Indeed, reading comprehension requires not only the integration of the processes necessary for decoding (e.g., transactions between perceptual stimuli), but also the combination of the phonological, orthographic, and semantic representations on the basis of the background information to create a proper understanding of the possible meanings of the text passage [74,75]. Nation and colleagues delve into the multifaceted nature of reading comprehension challenges, pinpointing insufficient vocabulary and sluggish reading pace as crucial contributing elements and examining the interplay between these factors across different reader profiles [76]. As texts become longer and more complex, cognitive demands increase, with the potential greater involvement of non-language-specific abilities [68,77,78]. In this regard, it has previously been observed that domain-general skills significantly contribute to reading comprehension, in addition to decoding and language skills [66,68,79,80,81]. For instance, when reading a text, domain-general processes are crucial for selecting and maintaining a coherent representation of the text [82,83,84,85,86], even after controlling for decoding and language skills [66,67,80].

Focusing on attentional control has been found to contribute significantly to comprehension proficiency, especially in upper-primary-grade students [85,87], because the more the decoding skills became consolidated, the more the executive resources could be dedicated entirely—or mostly—to the text’s comprehension. In addition, several cross-sectional and longitudinal studies have demonstrated the key role of attentional control in suppressing irrelevant information during text comprehension [59,61,82,88,89]. However, mixed results were found in other studies [68,90,91]. In this regard, Fenesi et al. [92] argued that researchers in education often overly focus on the short-term storage aspects of the Baddeley and Hitch [93] multicomponent model. They proposed a shift towards emphasizing attention control based on the executive attention view of individual differences in working memory [32] and long-term memory, according to the embedded process model [94,95] in educational research. Consistent with this, McVay and Kane [70] found in their research that mind wandering (i.e., the state where the default mode network predominates [96]) played a crucial mediating role in the connection between working memory capacity (WMC) and reading comprehension. This implies that the correlation between WMC and comprehension is influenced, at least partially, by the ability to keep attention away from intrusive thoughts.

In summary, it is widely recognized that attentional control is a crucial individual difference factor that significantly impacts various complex cognitive, academic, and related skills. However, the relations and interdependencies among attentional control and other crucial cognitive processes in the visuo-spatial and visuo-constructive domains with regard to both decoding and text comprehension are still unclear. Moreover, despite the large consensus on the importance of executive domain-general mechanisms in reading, relatively few studies have investigated their association with the different reading outcomes [14], and much uncertainty still exists about the relationship between attentional control and both lower-level (accuracy and speed of decoding) and higher-level (comprehension) reading. Specifically, the majority of studies have highlighted a link only to reading comprehension [66,81,91], while little is known about the effects of executive attention on the different types of reading outcomes [61,68].

### 1.4. Aims and Hypothesis

Building upon these premises, in this study, we investigated the relationship between reading performance, language, short-term and working memory, and visuo-spatial cognitive abilities using tests with different loads of attentional control. We used mixture modelling (latent profile analysis) to identify clusters of participants that differed quantitatively and qualitatively in either lower and higher levels of reading skills (i.e., word, non-word, text reading accuracy, and velocity). Then, we used this classification to investigate group differences in a variety of linguistic, working memory, and visuo-spatial tasks, as well as on reading comprehension skills, by mean of multivariate and univariate tests. Finally, we investigated the predictive power of different neuropsychological abilities on reading comprehension using dominance analysis. We hypothesize that good language abilities, higher attentional control, and visuo-spatial and visuo-constructive skills could drive proficient reading performance and reading comprehension.

## 2. Materials and Methods

### 2.1. Participants

The study involved 115 Italian fourth-grade students (61 males and 54 females) aged from 8 years and 6 months to 9 years and 10 months. Children were recruited through two primary schools in the urban area of a large northwestern Italian city. All the children were Italian native speakers with at least two years of literacy instruction and normal or correct-to-normal vision and hearing abilities. None of them had any psychological, neurological, or medical diagnoses, such as ADHD, autism spectrum disorder (ASD), epilepsy, or other relevant neurological and medical conditions. All the participants and their parents received a detailed explanation of the procedure and provided signed informed consent in accordance with the Declaration of Helsinki.

### 2.2. Procedure

All the children underwent a comprehensive neuropsychological battery (all the tests are listed in Table 1; see the Supplementary Materials for a detailed description of all the neuropsychological tests used in the study). Children were tested individually in a quiet room at their school. The battery of tests was administered on five different days, with each session lasting approximately one hour. The tests’ order was randomized between participants. For the purpose of the present study, we considered only a subset of 13 tests, namely the following:

- The language and executive attention domain: forward enumeration, rapid naming of colors, and verbal fluency (phonological);
- The short-term memory and working memory domain: digit span forward, digit span backward, alpha span, and updating of objects;
- The visuo-spatial and visuo-constructive domain: Rey figure (copy), TPV subtest copy, TPV subtest spatial position, and TPV subtest spatial relation, visuo-spatial span (Corsi test) forward, visuo-spatial span (Corsi test) backward.

Within each domain, tests are characterized by an increasing demand for attentional control.

### 2.3. Statistical Analysis

#### 2.3.1. Latent Profile Analysis (LPA)

We used latent profile analysis (LPA) to identify groups of patterns on reading ability tasks, namely speed (syllables per second) and accuracy (number of error) in reading words, pseudowords, and text, all expressed as Z-scores. LPA is a person-centered approach that focuses on relations among individuals in order to sort them into groups in which they are similar to each other and different from those in other groups [104]. In order to determine the most suitable number of classes for the sample, each model was assessed using the bootstrapped likelihood ratio test (BLRT; [105,106]), Akaike information criterion (AIC; [107]), Bayesian information criterion (BIC; [108]) and sample size-adjusted Bayesian information criterion (sBIC; [108]). The BLRT assesses the adequacy of a target model, such as a 2-class model, by comparing it to a comparison model that specifies one fewer class, such as a 1-class model. The *p*-value obtained from BLRT determines whether the solution with a greater number of classes (*p* < 0.05) or a lesser number of classes (*p* > 0.05) provides a better fit. The AIC and sBIC are fit indices that provide a description of how well a model fits the data. Smaller values of these indices imply a better fit of the model. It is important to mention that small classes, which make up less than 5% of the sample, are generally considered insignificant classes. This is often a result of extracting too many classes or profiles [109]. Therefore, when determining the ideal number of classes, class size was also taken into account. LPA was performed using Mplus 7 (Mplus User’s Guide, Seventh Edition, Los Angeles, CA, USA, 1998-2015).

#### 2.3.2. MANOVAs

To explore the multi-componentiality of reading abilities between groups, we conducted three multivariate analyses of variance (MANOVAs) using the cluster membership variable identified in Step 1 as an independent variable and three domains of neuropsychological tests as dependent variables (Domains A, B, and C hereafter).

In the first MANOVA (model 1), we compared the three groups of children on a cluster of 4 tests that assessed short-term memory and working memory abilities, i.e., digit span forward, digit span backward, alpha span, and updating of objects (Domain A).

In the second MANOVA (model 2), we compared the three groups of children on a cluster of 3 tests that assessed Language and executive attention, i.e., forward enumeration, rapid naming of colors, and verbal fluency (phonological) (Domain B).

In the third and last MANOVA (model 3), we compared the three groups of children on a cluster of 6 tests that assessed visuo-spatial and visuo-constructive abilities, i.e.,: visuo-spatial span (Corsi test) forward, visuo-spatial span (Corsi test) backward, Rey figure (copy), TPV subtest copy, TPV subtest spatial position, and TPV subtest spatial relation (Domain C).

The presence of multivariate outliers in the data was assessed using Mahalanobis distance. The multivariate normality was checked using a Shapiro–Wilk normality test, and homogeneity of variance–covariance matrices was assessed with Box’s M-test. When the MANOVA assumptions were not met, models were computed using nonparametric comparison of multivariate samples [110]. This method allowed us to perform analyses of one-way multivariate data using nonparametric techniques. It compares the multivariate distributions of the different samples and provides F-approximations and a permutation test for MANOVA type (Bartlett–Nanda–Pillai test statistics) as well as nonparametric relative effects. This analysis was performed using the npmv package (version 2.4.0; [111]) in R (R version 3.6.1 (2019-07-05), R Core Team, R Foundation for Statistical Computing, Vienna, Austria) [112].

When the MANOVA results were found to be significant, univariate analyses (ANOVAs) were performed in order to identify the specific dependent variables that contributed to the significant global effect. Univariate analyses were computed using a Welch ANOVA test.

When the ANOVA results were found to be significant, pairwise comparisons were performed to explore between-group differences (Table 5). Specifically, Games–Howell post hoc tests with Benjamini–Hochberg adjustment for *p*-values were used. Effect sizes were calculated for multivariate and univariate analyses, as well as for post hoc comparisons. 

All the R packages used to perform the analyses together with all the relative references are listed in the Supplementary Materials.

#### 2.3.3. ANOVA

To explore the differences between the comprehension ability in the three groups of children (as identified by LPA, according to the reading abilities), we performed a Welch ANOVA with reading comprehension score as the response variable and the LPA groups as the predictor. Levene’s test was used to assess homogeneity of variance across groups, and the Shapiro–Wilk test of normality was used to assess the normality of the ANOVA’s residuals. Games–Howell post hoc tests with Benjamini–Hochberg adjustment for *p*-values were also used. All the R packages used to perform the analyses together with all the relative references are listed in the Supplementary Materials.

#### 2.3.4. Dominance Analysis

In order to test the predictive power of language, executive, and visuo-spatial abilities on reading comprehension, we used dominance analysis (DA). DA is a statistical method for determining the relative importance of each predictor, when there is multicollinearity among predictors [113,114]. DA allows the full partition of the total variance explained by the predictors. Moreover, it provides the predictors’ dominance weights (which is an index of the predictors’ estimated importance) after an iterative process comparing predictors across different regression models. It also allows for the examination of different patterns of dominance, but in this study, we considered only general dominance (GA). GA provides information about the variance that a predictor explains when it is in combination with other predictors, and it is indexed by the Lindeman et al.’s (1980) R^2^ contribution averaged over orderings among regressors. The theoretical distribution of dominance weights is unknown, therefore we used bootstrapping to capture random component of the regression models and obtain confidence intervals for testing whether a dominance weight is different from zero, as well as for comparing dominance weights in the same model [113]. To analyze data, we used the relaimpo (version 2.2-6; [115]) package in R [112]. All the R packages used to perform the analyses together with all the relative references are listed in the Supplementary Materials.

## 3. Results

### 3.1. LPA

**Figure 1:**
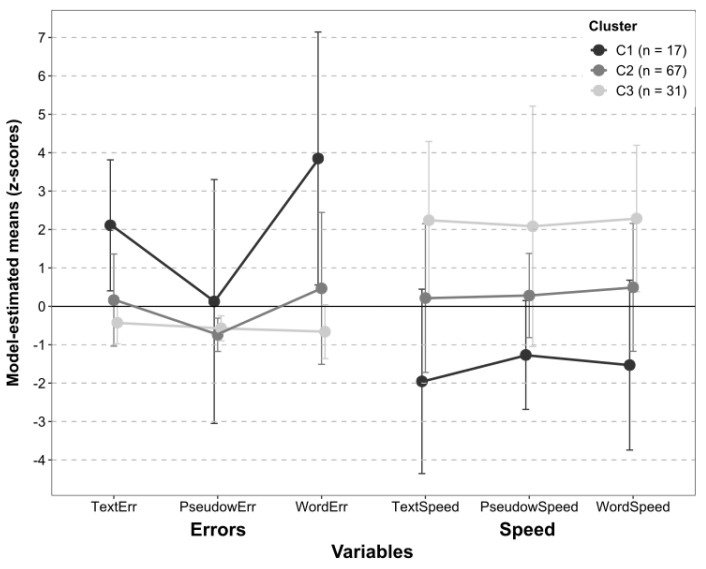
The figure represents the mean Z-score estimated by the latent profile analysis (LPA) model. The six reading measures included as variables in the LPA are shown in the x axis; the three groups of children—as clustered by the LPA results—are represented by the lines in grayscale, namely the ‘poor readers’ (C1, *N* = 17) in dark grey, the ‘average readers’ (C2, *N* = 67) in medium grey, and the ‘good readers’ (C3, *N* = 31) in light grey. Vertical bars represent the standard errors.

We could test the fit of LPA models with two and three clusters, as with four clusters, we encountered estimation problems. According to the criteria described above, we chose the three-cluster model (AIC = 2264.291, BIC = 2335.659, SBIC = 2253.478, *p*(BLRT) < 0.001) over the two-cluster model (AIC = 2359.121, BIC = 2411.274, SBIC = 2351.219). Based on children’s profile scores, clusters were labelled as ‘poor readers’ (C1, *N* = 17), ‘average readers’ (C2, *N* = 67), and ‘good readers’ (C3, *N* = 31) (Figure 1). Table 2 shows the estimated means for each variable, their 95% confidence intervals, and the post hoc comparisons between the three clusters of children (Benjamini–Hochberg correction; [116]).

### 3.2. MANOVAs

The assumption of the homogeneity of variance–covariance matrices was met in model 1, but not in model 2 and 3 (see Table 3). The assumption of multivariate normality was not met. Therefore, for model 2 and 3, we used a nonparametric estimation of the effects, and the comparisons were performed using a permutation test for Nanda–Pillai test statistics.

**Figure 2:**
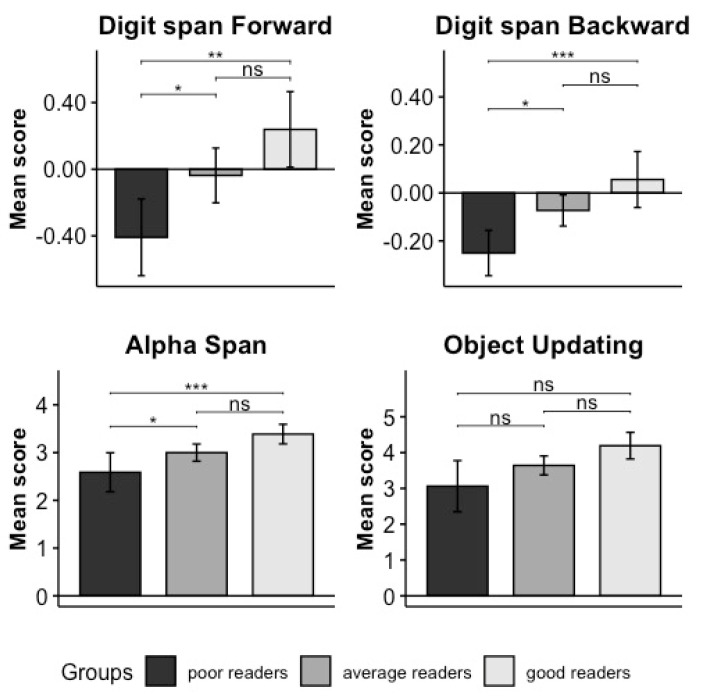
Results of the univariate post hoc comparison performed in Domain A. The name of every specific test is indicated on the top of each plot, and the mean of each test score is presented on the Y axis (z scores in digit span forward and backward and number of correctly recalled elements in alpha span and updating of object). The significance levels of results are indicated as follows: ‘***’ means *p* = 0.001, ‘**’ means *p* = 0.01, ‘*’ means *p* = 0.05, and “ns” means non-significant. Poor readers are represented in dark grey, good readers in light grey, and average readers in medium–dark grey. Vertical bars represent standard errors.

Analyses on Domain A: The result of the MANOVA was significant (F_(8,221)_ = 3.449, *p* = 0.001, permutation *p* = 0.001; η^2^_G_ = 0.183 [0.07–0.33]). All the univariate analyses revealed significant results for all the variables (see Table 4). Post hoc comparisons (Table 5 and Figure 2) showed that good readers outperformed poor readers in digit span forward (*p* = 0.005), digit span backward (*p* = 0.002), alpha span (*p* < 0.001), and object updating (*p* = 0.003). Moreover, the average readers performed significantly better than the poor readers in alpha span (*p* = 0.03) and marginally better in object updating (*p* = 0.06) (the mean and SD are reported in Table 6).

**Figure 3:**
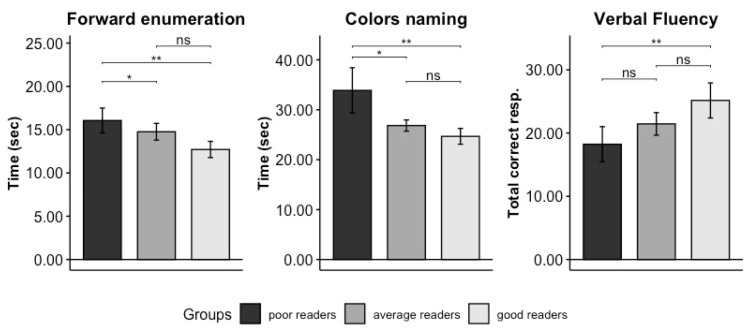
Results of the univariate post hoc comparison performed in Domain B. The name of every specific test is indicated at the top of each plot, and the means of each test score—namely time (in seconds) in forward enumeration and color naming and number of correctly recalled elements in verbal fluency—are presented on the Y axis. The significance levels of results are indicated as follows: ‘**’ means *p* = 0.01, ‘*’ means *p* = 0.05, and “ns” means non-significant. Poor readers are represented in dark grey, good readers in light grey, and average readers in medium–dark grey. Vertical bars represent standard errors.

Analyses of Domain B: The result of the MANOVA was significant (F_(6.159,225.911)_ = 5926, *p* < 0.001, permutation *p* < 0.001, η^2^_G_ = 0.238 [0.108–0.387]). Since the normality and homogeneity of variance assumptions were not met (Table 3), the univariate analyses were computed using a Welch ANOVA test. Univariate ANOVAs revealed significant results for all the variables considered (see Table 4). The results of post hoc comparisons (Games–Howell test, Table 5 and Figure 3) in the forward enumeration task showed that good readers performed significantly faster than average (*p* = 0.012) and poor readers (*p* = 0.002), while no difference was found between poor and average readers (*p* = 0.277). Results of post hoc comparisons in the color-naming test showed that poor readers group performed significantly slower than average readers (*p* = 0.013) and good readers (*p* = 0.002). Finally, the results of post hoc comparison of verbal fluency showed that the poor readers performed more poorly than the good readers (*p* = 0.002); no other differences were found to be significant (the mean and SD are reported in Table 6).

**Figure 4:**
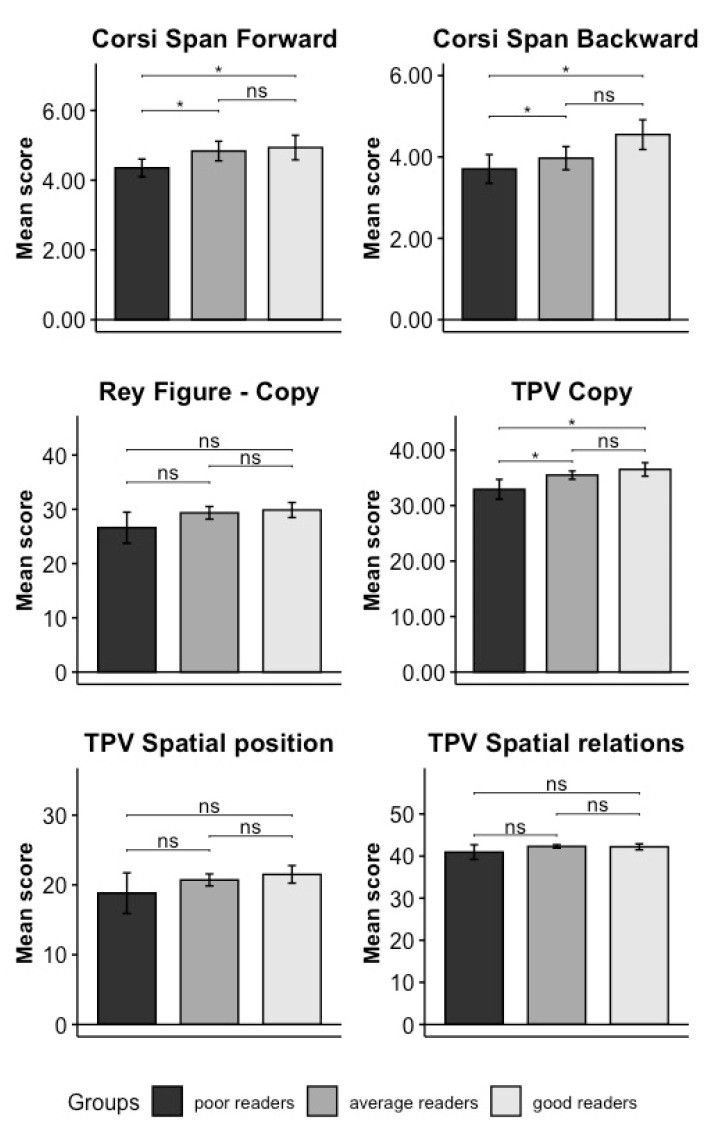
Results of the univariate post hoc comparison performed in Domain C. The name of every specific test is indicated on the top of each plot, and the mean of each test score—namely the number of elements correctly recalled in the forward and backward Corsi test, the mean of the total score in the Rey figure, and the mean of each of the TPV tests—are presented on the Y axis. The significance levels of the results are indicated as follows: ‘*’ means 0.01 < *p* < 0.05, and “ns” means non-significant (*p* > 0.05). Poor readers are represented in dark grey, good readers in light grey, and average readers in medium–dark grey. Vertical bars represent standard errors.

**Analyses of Domain C**: The MANOVA result was significant (F_(12.32,217.75)_ = 1.838, *p* = 0.042, permutation *p* = 0.035, η^2^_G_ = 0.143 [0.038–0.289]). Since the normality and homogeneity of variance assumptions were not met (Table 3), the univariate analyses were computed using the Welch ANOVA Test. ANOVAs revealed significant results in both forward and backward Corsi tests and the TPV subtest copy (see Table 4 and Figure 4). Post hoc comparisons (Games–Howell test, Table 5 and Figure 4) in the forward Corsi test showed lower performance in poor readers compared to average (*p* = 0.29) and good readers (*p* = 0.022). Similarly, in the TPV subtest copy, the performance of poor readers was lower than that of average readers (0.029) and good readers (0.004). Finally, multiple comparisons in the backward Corsi test showed that the group of good readers outperformed the group of average (0.036) and poor readers (0.004). No other difference was found to be significant.

### 3.3. ANOVA

Within the ANOVA results on reading comprehension, assumption of the homogeneity of variance was met (F_(2,41.06)_ = 1.6689 *p* = 0.193), while the Shapiro–Wilk normality test showed significant results (W = 0.959, *p*-value = 0.001). Therefore, we further inspected the residual distribution compared to the theoretical normal distribution by means of a quantile–quantile plot. QQ plots showed slightly tailed distribution, most likely due to small sample size. Since the ANOVA is considered a robust test in case of violations of the assumption of normality, we decided to perform the analysis using a Welch ANOVA. The Welch ANOVA results showed a significant effect by group (F_(2,41.06)_: 39,65, *p* < 0.001). Games–Howell post hoc comparisons showed that comprehension ability was greater in good readers compared to the average (*p* < 0.001) and poor readers (*p* < 0.001), and it was also greater in average readers compared to poor readers (*p* < 0.001).

### 3.4. Dominance Analysis

Results (Table 7) showed that reading comprehension was significantly positively associated with working memory (alpha span, digit span forward), verbal fluency skills and text-reading speed. These predictors accounted for 6%, 5%, 4%, and 8% of variance, respectively.

## 4. Discussion

In this study, we investigated the relationship between reading performance (in terms of both decoding and comprehension) and three domains of neuropsychological abilities, namely language, short-term and working memory, and visuo-spatial skills. We used mixture modelling (latent profile analysis) to identify clusters of participants that differed quantitatively in their reading skills (i.e., word, non-word, text reading, and text comprehension), and investigated group differences on a variety of neuropsychological tests, which were organized in the three above-mentioned domains. According to this framework, reading efficiency is intricately linked to the proper functioning of individual sub-components and the “central processor” [117,118] responsible for allocating necessary attentional resources for assembling these components [119].

### 4.1. Reading—Decoding

Our study contributed to the understanding of decoding processes by revealing significant associations with attentional control mechanisms. Extensive research underscores the role of language processes such as phonological awareness and rapid naming in decoding speed and accuracy [43,44,45,120]. This study uncovered intriguing insights: good readers consistently outperformed poor readers across all three domains (linguistic, visuo-spatial, and executive attention). Regarding the domain of attentional control and working memory, a distinction emerged between good and poor readers across short-term and working memory tasks (i.e., digit span forward, digit span backward, alpha span), indicating a positive correlation between working memory capacity (WMC) and reading fluency. This finding aligns with previous research highlighting the importance of working memory resources (particularly verbal working memory) in automating reading processes [61,68,69]. However, the findings discussed in this paper also align with the less commonly referenced literature that suggests correlations between decoding and the visuo-spatial domain [56].

Furthermore, attentional control emerges as a critical determinant of reading proficiency alongside language skills. Our study underscores the intertwined nature of attentional control and decoding by revealing significant discrepancies between good and poor readers in language-related tasks, where there is a progressively heightened involvement of attentional control. (i.e., forward enumeration, color naming, and verbal fluency). This suggests that attentional mechanisms not only facilitate decoding processes but also influence higher-level linguistic processes. Indeed, attentional control allows readers to flexibly place focus upon relevant information while ignoring distractions [22,27,40]. This ability is crucial for decoding as it facilitates the sequential processing of graphemes within words [41,44,45]. Additionally, attentional control enables the efficient extraction of various types of information from a string of letters, including orthographic, phonological, and morphological representations [49,50]. This flexibility is particularly relevant for languages with opaque orthography [51] and plays a vital role in managing lexical retrieval processes [49].

In addition to language, working memory and attentional control, our research shed light on the influence of visuo-spatial and visuo-constructive skills on reading performance. Good readers exhibited superior performance in visuo-spatial tasks (Corsi test-forward and backward, TPV subtest copy) compared to poor and average readers, indicating a potential link between visuo-spatial abilities and reading proficiency. This observation contributes valuable evidence to the growing body of literature highlighting the significance of visuo-spatial processing in literacy development [46,47,48]. It supports the emergence of a multi-componential model of reading, as evidenced by multiple studies [14,36,115].

### 4.2. Reading—Comprehension

Reading comprehension ability was found to be significantly higher in proficient readers compared to average and poor readers, with a marked difference also between average and poor readers. This gradation in comprehension skills underscores the impact of working memory, verbal fluency, and reading speed on reading proficiency, as shown by our dominance analysis.

The importance of verbal fluency, in particular, is underscored by its association with the ability to swiftly access and utilize linguistic information, which is critical for understanding and integrating text [14,121,122]. Furthermore, our results align with evidence suggesting that proficiency in working memory tasks is closely related to reading comprehension skills in children (e.g., [123,124]). A distinct verbal short-term memory, as part of the working memory capacity (WMC) model described by Engle and Kane [125], appears beneficial during the “construction” phase previously mentioned, in addition to having an adequate reading speed.

The role of working memory in reading comprehension is multifaceted, extending beyond mere storage, which is primarily the purview of short-term memory. It encompasses not only the temporary storage but also the manipulation and ongoing maintenance of information to facilitate complex cognitive tasks [126]. Interestingly, increased working memory demands can impair the integration of information across different text parts or the detection of inconsistencies within texts, particularly in poor comprehenders [67,127]. Moreover, the critical function of recall in accessing stored words and their meanings highlights the integral role of verbal fluency in reading comprehension.

Effective reading comprehension, as our findings suggest, is predicated on a synergistic interplay of attentional control, working memory, reading speed, and verbal fluency. Attentional control, in particular, orchestrates these cognitive processes, massively influencing reading comprehension [82,88,89]. Carretti et al. [82] further elucidate this by demonstrating that verbal memory tasks requiring high attentional control can effectively distinguish between poor and good comprehenders, indicating that both domain-specific and general factors contribute to reading comprehension performance. This evidence aligns with our results, which support the notion that higher processing efficiency allows for an optimal allocation of cognitive resources, thereby enhancing comprehension.

Our study’s insights into the specific set of domain-general skills account for 23% of the variance in reading comprehension compared to the 62% explained by the entire model, highlighting the significant but partial contribution of these factors within the broader reading process. This opens avenues for targeted interventions, as evidenced by the success of the integrated cognitive training approach [37,128], which addresses comprehension challenges through methods designed to enhance reading speed, vocabulary, verbal fluency, and attentional control. These interventions are grounded in a protocol that reflects our findings, underscoring the importance of a multifaceted approach to improving reading skills.

## 5. Study Limitations and Further Research

Our study presents several limitations that highlight areas for improvement in future research. Firstly, our research focused exclusively on fourth-grade Italian-speaking children. This narrow demographic focus restricts the generalizability of our findings to other age groups and language backgrounds. Indeed, Italian is a very regular language, characterized by a consistent correspondence between orthography and phonology. Therefore, it is possible that reading decoding and comprehension in languages with less transparent orthography may recruit different cognitive skills. Thus, including participants from a more diverse range of linguistic backgrounds could provide a deeper understanding of how specific language characteristics, such as orthographic transparency, influence reading performance.

Additionally, the relationship between reading performance and executive components may not be uniform across different age groups and levels of reading proficiency. It is essential for future studies to explore these potential variations to offer a more detailed insight into the cognitive mechanisms underpinning reading comprehension.

Another limitation concerns the observed lack of automaticity in the reading process, which may be associated with the executive resources demanded by reading tasks. Notably, the decoding-based reading system continues to develop until adolescence. The nature of this relationship, along with its implications for reading intervention strategies, warrants further exploration to clarify more precisely the role of executive attention in reading development.

Finally, we explored comprehension abilities only by means of text reading. However, comprehension performance may vary between reading and listening modalities. Therefore, forthcoming studies ought to explore the potential discernment of distinct profiles within the cohort of poor readers, based on their achievements in reading and listening comprehension.

In light of these limitations, several directions for future research emerge. There is a critical need for studies that aim to validate and generalize the findings of our study across a broader range of populations. Such research endeavors would not only augment the theoretical framework surrounding reading comprehension but also have practical implications for educational and clinical interventions.

## 6. Conclusions

Overall, our study contributes to the understanding of decoding and comprehension processes. Specifically, our study emphasizes the interconnectedness of attentional control, working memory capacity (WMC), and decoding, while also confirming the well-established correlation between decoding proficiency and language proficiency. Furthermore, we concur with the growing, albeit less frequently acknowledged, body of literature that underscores the importance of visuo-spatial processing in the development of literacy.

In terms of comprehension, our findings indicate that proficient reading comprehension relies on a synergistic interaction between attentional control, working memory, reading speed, and verbal fluency. Collectively, our results endorse the emergence of a multi-componential model of reading. This underscores the significance of adopting a comprehensive approach to enhancing reading abilities and paves the way for targeted interventions. Such interventions should aim to address comprehension difficulties by enhancing reading speed, expanding vocabulary, improving verbal fluency, and refining attentional control.

## Figures and Tables

**Table 1:** List of the tests administered in the present study. Please see the Supplementary Materials for detailed descriptions.

Language and Executive Attention Domain	Short-Term Memory and Working Memory Domain	Visuo-Spatial and Visuo-Constructive Domain
Forward enumeration (MEA; [97])	Digit span—Forward and Backward (BVN; [98])	Rey’s Figure—Copy [99]
Rapid naming of colours (MEA; [97])	Alpha span (MEA; [97])	Developmental Visual Perception Test-TPV [100]
Verbal fluency (CMF Battery; [101])	Object Updating (adapted from [102])	Corsi’s Test (Forward and backward; [103])

**Table 2:** Estimated means for each LPA variable (in rows), the relative 95% confidence intervals, and the post hoc comparisons between the three clusters of children (in columns).

Variable	C1 (*n* = 17)	C2 (*n* = 67)	C3 (*n* = 31)	Post Hoc
TextErr	2.11 [0.40; 3.81]	0.16 [−1.04; 1.36]	−0.43 [−0.98; 0.13]	C1 > C2; C1 > C3
TextSpeed	−1.95 [−4.35; 0.45]	0.22 [−1.72; 2.15]	2.24 [0.19; 4.29]	C3 > C2 > C1
PseudowErr	0.13 [−3.05; 3.30]	−0.74 [−1.17; −0.31]	−0.57 [−0.90; −0.25]	None
PseudowSpeed	−1.27 [−2.68; 0.15]	0.28 [−0.81; 1.38]	2.08 [−1.05; 5.21]	C3 > C1; C2 > C1
WordErr	3.85 [0.56; 7.14]	0.47 [−1.51; 2.44]	−0.66 [−1.36; 0.04]	C1 > C2; C1 > C3
WordSpeed	−1.53 [−3.74; 0.68]	0.49 [−1.17; 2.16]	2.29 [0.38; 4.20]	C3 > C2 > C1

**Table 3:** Results of Box’s M tests and Shapiro tests for multivariate normality in the four MANOVA models.

	Box’s M Tests			Shapiro Test for Multivariate Normality	
	Statistic	*p*-Value	Parameter	Statistic	*p*-Value
Model1	27.00	0.135	20	0.91	<0.001
Model2	39.30	<0.001	12	0.92	<0.001
Model3	86.45	<0.001	42	0.88	<0.001

**Table 4:** Results of all univariate ANOVAs; effect sizes are reported in the last column together with the 95% CI within squared brackets.

Domain	Task	*n*	F	DFn	DFd	*p*	Method	η^2^ [CI 95%]
Domain A	Digit Span Forward	115	8.67	2	47.7	<0.001	Welch ANOVA	0.27 [0.06; 0.43]
	Digit Span Backward	115	9.63	2	46.2	<0.001	Welch ANOVA	0.29 [0.08; 0.46]
	Alpha Span	115	8.09	2	40.6	0.001	Welch ANOVA	0.28 [0.06; 0.46]
	Object Updating	115	5.30	2	36.4	0.010	Welch ANOVA	0.23 [0.02; 0.41]
Domain B	Forward Enumeration	115	9.54	2	46.4	<0.001	Welch ANOVA	0.29 [0.08; 0.45]
	Color Naming	115	8.72	2	35.9	<0.001	Welch ANOVA	0.33 [0.07; 0.50]
	Verbal Fluency	115	6.64	2	45.3	0.003	Welch ANOVA	0.23 [0.03; 0.40]
Domain C	Corsi Forward	115	5.26	2	58.1	0.008	Welch ANOVA	0.15 [0.01; 0.30]
	Corsi Backward	115	6.11	2	50.6	0.004	Welch ANOVA	0.19 [0.02; 0.36]
	Rey Figure—Copy	115	2.31	2	39.7	0.112	Welch ANOVA	0.10 [0.00, 0.27]
	TPV—Copy	115	5.94	2	38.7	0.006	Welch ANOVA	0.24 [0.03; 0.41]
	TPV—Spatial Position	115	1.69	2	36.4	0.199	Welch ANOVA	0.09 [0.00, 0.25]
	TPV—Spatial Relation	115	1.27	2	34.0	0.293	Welch ANOVA	0.07 [0.00, 0.23]

**Table 5:** Results of univariate post hoc comparisons (Games–Howell post hoc test with Benjamini–Hochberg adjustment for *p*-values). In each cluster, post hoc comparisons were performed only when the univariate ANOVA produced significant results.

		Comparison	Mean Difference [95% CI]	t	df	*p*-Value	p.adj (Benjamini–Hochberg)	Cohen’s d [95% CI]
Domain A	Digit Span Forward	2-1	0.37 [0.04; 0.70]	2.72	36.68	0.026	0.039	1.12 [0.56; 1.67]
		3-1	0.65 [0.27; 1.03]	4.17	42.28	0.000	0.001	1.34 [0.68; 1.99]
		3-2	0.28 [−0.06; 0.61]	2.00	63.28	0.122	0.122	0.54 [0.11; 0.97]
	Digit Span Backward	2-1	0.18 [0.04; 0.31]	3.22	35.17	0.008	0.011	1.35 [0.78; 1.92]
		3-1	0.31 [0.13; 0.48]	4.23	45.82	0.000	0.001	1.31 [0.65; 1.95]
		3-2	0.13 [−0.03; 0.29]	1.96	50.17	0.133	0.133	0.60 [0.16; 1.03]
	Alpha Span	2-1	0.41 [−0.12; 0.94]	1.93	23.50	0.151	0.151	0.99 [0.43; 1.54]
		3-1	0.80 [0.26; 1.34]	3.67	24.85	0.003	0.010	1.54 [0.86; 2.20]
		3-2	0.39 [0.06; 0.71]	2.87	75.68	0.015	0.022	0.71 [0.27; 1.15]
	Object Updating	2-1	0.58 [−0.33; 1.49]	1.61	19.93	0.265	0.265	0.90 [0.35; 1.44]
		3-1	1.13 [0.18; 2.08]	2.97	24.12	0.018	0.053	1.26 [0.61; 1.90]
		3-2	0.55 [0.01; 1.09]	2.45	62.16	0.044	0.067	0.67 [0.23; 1.10]
Domain B	Forward Enumeration	2-1	−1.30 [−3.34; 0.74]	1.56	34.32	0.277	0.277	0.66 [0.12; 1.20]
		3-1	−3.35 [−5.37; −1.33]	4.09	30.64	0.001	0.002	1.54 [0.87; 2.21]
		3-2	−2.05 [−3.64; −0.46]	3.07	85.61	0.008	0.012	0.71 [0.27; 1.15]
	Color Naming	2-1	−7.05 [−12.69; −1.40]	3.18	18.27	0.013	0.020	1.85 [1.24; 2.45]
		3-1	−9.20 [−14.96; −3.45]	4.04	20.26	0.002	0.005	1.88 [1.17; 2.57]
		3-2	−2.16 [−4.46; 0.14]	2.25	62.32	0.070	0.070	0.61 [0.18; 1.05]
	Verbal Fluency	2-1	3.21 [−0.68; 7.11]	2.02	32.68	0.122	0.122	0.88 [0.33; 1.43]
		3-1	6.93 [2.34; 11.51]	3.67	42.63	0.002	0.006	1.18 [0.53; 1.81]
		3-2	3.71 [−0.20; 7.63]	2.28	56.79	0.067	0.100	0.65 [0.21; 1.08]
Domain C	TPV—Copy	2-1	2.51 [0.20; 4.83]	2.72	22.91	0.032	0.047	1.41 [0.84; 1.99]
		3-1	3.57 [1.04; 6.11]	3.47	31.76	0.004	0.013	1.29 [0.63; 1.93]
		3-2	1.06 [−0.64; 2.76]	1.50	55.07	0.297	0.297	0.43 [0.00; 0.86]
	Corsi Forward	2-1	0.48 [0.04; 0.93]	2.62	62.04	0.029	0.044	0.83 [0.28; 1.37]
		3-1	0.58 [0.07; 1.09]	2.77	45.87	0.022	0.044	0.86 [0.23; 1.47]
		3-2	0.10 [−0.43; 0.63]	0.45	68.91	0.896	0.896	0.12 [−0.31; 0.54]
	Corsi Backward	2-1	0.26 [−0.27; 0.80]	1.20	42.71	0.459	0.459	0.46 [−0.08; 0.99]
		3-1	0.84 [0.25; 1.43]	3.45	43.40	0.004	0.011	1.09 [0.46; 1.72]
		3-2	0.58 [0.03; 1.13]	2.53	67.98	0.036	0.055	0.66 [0.22; 1.09]

**Table 6:** Table showing the mean and standard deviation (column 5 and 6, respectively) of the scores obtained in all the neuropsychological tests (column 3 “Variable”) by the three groups of children (column 2 “Cluster”) defined though latent profile analysis. The numerosity of each group is indicated in column 4 (“*N*”). The first column (“Domain”) indicates the three domains of neuropsychological tests used in the MANOVA analyses.

Domain	Cluster	Variable	*N*	Mean	Sd
Domain A	1	Digit Span Forward	17	−0.409	0.448
	2	Digit Span Forward	67	−0.037	0.674
	3	Digit Span Forward	31	0.239	0.619
	1	Digit Span Backward	17	−0.251	0.184
	2	Digit Span Backward	67	−0.073	0.266
	3	Digit Span Backward	31	0.056	0.318
	1	Alpha Span	17	2.588	0.795
	2	Alpha Span	67	3.000	0.739
	3	Alpha Span	31	3.387	0.558
	1	Object Updating	16	3.062	1.340
	2	Object Updating	67	3.642	1.083
	3	Object Updating	31	4.194	1.014
Domain B	1	Color Naming	17	33.882	8.831
	2	Color Naming	67	26.836	4.614
	3	Color Naming	31	24.677	4.308
	1	Forward Enumeration	17	16.059	2.794
	2	Forward Enumeration	67	14.761	3.962
	3	Forward Enumeration	31	12.710	2.559
	1	Verbal Fluency	17	18.235	5.403
	2	Verbal Fluency	67	21.448	7.324
	3	Verbal Fluency	31	25.161	7.568
Domain C	1	Corsi Forward	17	4.353	0.493
	2	Corsi Forward	67	4.836	1.149
	3	Corsi Forward	31	4.935	0.964
	1	Corsi Backward	17	3.706	0.686
	2	Corsi Backward	66	3.970	1.163
	3	Corsi Backward	31	4.548	0.995
	1	TPV—Spatial Rapresentation	17	40.941	3.381
	2	TPV—Spatial Rapresentation	67	42.299	1.596
	3	TPV—Spatial Rapresentation	31	42.194	1.939
	1	Rey Figure—Copy	17	26.618	5.547
	2	Rey Figure—Copy	67	29.351	4.776
	3	Rey Figure—Copy	31	29.871	3.755
	1	TPV—Spatial Position	17	18.824	5.659
	2	TPV—Spatial Position	67	20.716	3.520
	3	TPV—Spatial Position	31	21.516	3.434
	1	TPV—Copy	17	32.941	3.473
	2	TPV—Copy	67	35.493	3.072
	3	TPV—Copy	31	36.516	3.315

**Table 7:** Results of dominance analysis performed on reading comprehension abilities. The tests used as predictors are indicated in the first column (“Predictors”), the coefficient of the association between each test and the reading comprehension is indicated in the second column (“Coef”), and asterisks indicate significance (“*” means *p* < 0.05). The value in the third column (“R^2^”) expresses the general dominance statistic value as a percentage of the overall fit statistic value; 95% confidence intervals are reported in squared brackets.

Predictor	Coef	R2
Alpha Span	0.11 *	0.05 [0.00, 0.09]
Digit Span Forward	0.24 *	0.03 [0.00, 0.06]
Digit Span Backward	0.43	0.04 [0.00, 0.08]
Updating of Objects	0.24	0.07 [0.00, 0.14]
Forward Enumeration	0.03	0.00 [−0.01, 0.01]
Rapid Naming of Colors	0.02	0.03 [−0.01, 0.06]
Verbal Fluency	0.03 *	0.06 [0.00, 0.10]
Visuo-spatial Span (Corsi Test) Forward	−0.07	0.01 [−0.01, 0.02]
Visuo-spatial Span (Corsi Test) Backward	0.08	0.02 [−0.01, 0.04]
TPV Subtest Spatial Relation	0.00	0.01 [−0.02, 0.03]
Rey Figure (Copy)	0.04	0.04 [−0.01, 0.09]
TPV Subtest Spatial Position	0.07	0.06 [−0.02, 0.12]
TPV Subtest Copy	−0.01	0.01 [−0.01, 0.03]
Text—Speed	0.23 *	0.08 [0.02, 0.14]
Text—Errors	−0.05	0.03 [−0.01, 0.05]
R^2^ tot		0.62 [0.50, 0.73]

## Data Availability

Data and R scripts used are available on OSF: https://osf.io/uvawz/?view_only=1a7d12293c094aadb85b4cb230ed4494 (accessed on 14 April 2024).

## Supplementary Materials

The following supporting information can be downloaded at: https://www.mdpi.com/article/10.3390/brainsci14040390/s1, in the supplementary materials we have inserted a detailed description of all the neuropsychological tests used in the present study (Supplementary Materials S1), a list of all the R packages use to perform the analyses together with all the relative references (Supplementary Materials S2).
