# Supplementary material for: Reading Skill Profiles in School-Aged Italian-Speaking Children: A Latent Profile Analysis Investigation into the Interplay of Decoding, Comprehension and Attentional Control
Source: Brain Sci. 2024 Apr 17;14(4):390. doi: 10.3390/brainsci14040390 (PMC11048682; doi:10.3390/brainsci14040390)
Supplement: Supplementary file 1 [file brainsci-14-00390-s001.zip › brainsci-2946714-supplementary.pdf]

Reading Skill Profiles in School-Aged Italian-Speaking Children: A Latent Profile Analysis Investigation into the Interplay of Decoding, Comprehension and Attentional Control

List of Supplementary materials:

- **Supplementary material S1:** detailed description of all the neuropsychological tests used in the present study.
- **Supplementary material S2:** list of the R packages used to perform the analyses, with relative refences.

Supplementary material S1

Here below we describe all the neuropsychological tests used in the present study:

- *Enumeration task – forward*, from the "Measure for the executive attention Battery - MEA " (Benso et al., 2019); and the Calculation switching task (Benso et al., 2005). The task requires counting from 1 to 100, the amount of time needed by the child to count is measured; this task implies skills more related to the linguistic components.

- *Digit Span - forward and backwards*, from the "Neuropsychological Battery for children aged 5-11 - BVN" (Bisiacchi, Cendron & Gugliotta, 2005). This is a verbal short-term memory task and included two parts: children were asked to listen to a list of numbers, which length progressively increased, and to repeat the sequence of number in the same (part A – Digit span Forwards) or reversed (part B – Digit span Backwards) order. The part B requires a higher involvement of working memory abilities. If the child was successful in repeating at least two of the three trials, a sequence one digit longer was presented and the task continued until a maximum of nine digits have been presented, or until two trials of the same block were wrong.

- *Alpha span task*, from the "Measure for the executive attention Battery - MEA " (Benso et al., 2019). The children were presented with a list of words and they were asked first to repeat the words in the same order as they were presented (passive span), and then to repeat the listened words in alphabetical order (active span). In particular, this task was indicated by Cowan et al. (2005) to evaluate the Working Memory Capacity (WMC), defined as a group of non-separable executive functions called "Executive Attention" (Engle, 2002). The so defined WMC includes all the processes responsible for the simultaneous storage and manipulation of information, with minimum or null

involvement of the chunking or repeating strategies. This models of the WCM is also in agreement with the revised model of Baddeley (Hofmann, 2011).

- *Object Updating task* (Palladino et al., 2001, revised by Benso et al., 2013). The children listened several lists of words, formed by abstract and concrete words, and were asked i) to repeat only the three words corresponding to tools, animals, or vegetables and ii) immediately after, to repeat only the name of two smaller objects. For instance, if the list presented was “hammer”, “elephant”, “anxiety”, “carrot”, “happiness”, the child was asked to first repeat “hammer”, “elephant”, “carrot” in the same order as they were presented, and then to repeat “carrot”, “hummer” that are the smallest ones. The first task involved auditory attention and short-term memory, while the second task involves also imaginative and working memory skills. The lists were grouped in blocks of three lists with the same length (i.e. number of words). If the child responded correctly, the length of the list in the subsequent block increased by one word. The task proceeded until the child responded correctly in two out of three lists of the same length in the same block. The number of words in the last list repeated correctly was considered.

- *Rapid naming of colors task* from the “Measure for the executive attention Battery - MEA ” (Benso et al.,2019). The children were asked to say aloud the name of 35 little colored circles (blue, yellow, green, red, black) presented on a vertical page. The total response time and number of errors were measured. These tasks measured the rapidity in retrieving the linguistic label of familiar stimuli (i.e., efficiency of lexical access).

- *Verbal fluency task* from the “Metaphonological awareness battery - CMF” (Marotta, Trisciani & Vicari, 2008). The children were asked to say aloud as many words as possible that pertain to a specific category (e.g. animals). The time limit was one minute for each category. The total number of correct words was measured. This task has been proposed to measure inhibitory functioning (Mahone, Koth, Cutting, Singer, & Denckla, 2001), memory monitoring (Rosen & Engle, 1997), and switching between retrieval strategies (Troyer, Moscovitch, & Winocur, 1997).

- *“Developmental Visual Perception Test - TPV”* (Hammill, Pearson, & Voress, 2003), the battery includes several tasks aimed to assess the visual perception and the visuo-motor integration. In particular, in the present study we used the substest TPV subtest copy, TPV subtest spatial position, and TPV subtest spatial relation. In “*Copy of figures*” task the children were asked to copy a number figures which complexity increased progressively; the “*Position in the space*” is a sort of match to sample task; finally in “S*patial relations*” task the children were presented with a number of points placed at a regular distance, some of which were connected with lines, then the children

were presented with the same matrix of points and were asked to draw the line to reproduce the same figure seen before.

- Corsis Test, - forward and backwards, from the Battery for the assessment of the visuo-spatial memory-BVS" (Mammarella, Toso, Pazzaglia & Cornoldi, 2008). In this task, the children were presented with several visuo-spatial sequences of increasing length and were asked to reproduce them in the same (part A) or reversed order (part B). The part B required a stronger involvement of the visuo-spatial working memory. This procedure was used for sequences of increasing length (up to a maximum of 9 digits).

- The *Reys Figure* (Lis & Di Nuovo, 1982) was used to assess the visual organization and the visuoconstructive functions. In this task, participants were asked to copy a complex figure composed of several geometrical elements.

Supplementary material S2

R packages used to perform the analyses:

npmv : Woodrow W. Burchett, Amanda R. Ellis, Solomon W. Harrar, Arne C. Bathke (2017). Nonparametric Inference for Multivariate Data: The R Package npmv. Journal of Statistical Software, 76(4), 1-18. doi:10.18637/jss.v076.i04

emmeans: Russell Lenth (2020). emmeans: Estimated Marginal Means, aka Least-Squares Means. R package version 1.5.1. https://CRAN.R-project.org/package=emmeans

rpsychi : Yasuyuki Okumura (2012). rpsychi: Statistics for psychiatric research. R package version 0.8. https://CRAN.R-project.org/package=rpsychi

heplots : John Fox and Michael Friendly and and Georges Monette (2020). heplots: Visualizing Tests in Multivariate Linear Models. R package version 1.3-7. URL

https://CRAN.R-project.org/package=heplots

To refer to the theory on which this package is based: Friendly, M. (2007). HE plots for Multivariate General Linear Models. Journal of Computational and Graphical Statistics,2007, 16, 421-444

For use with repeated measures designs: Michael Friendly (2010). HE Plots for Repeated Measures Designs.
Journal of Statistical Software, 37(4), 1-40. URL
https://www.jstatsoft.org/v37/i04/.

userfriendlyscience : Peters G (2018). _userfriendlyscience: Quantitative analysis made
accessible_. doi: 10.17605/osf.io/txequ (URL:
https://doi.org/10.17605/osf.io/txequ), R package version 0.7.2,
<URL: https://userfriendlyscience.com>.

Peters G (2017). "Diamond plots: a tutorial to introduce a
visualisation tool that facilitates interpretation and comparison
of multiple sample estimates while respecting their inaccuracy."
PsyArXiv. <URL: https://psyarxiv.com/fzh6c>.

MBESS : Ken Kelley (2020). MBESS: The MBESS R Package. R package version
4.8.0. https://CRAN.R-project.org/package=MBESS

MOTE : Buchanan E, Gillenwaters A, Scofield J, Valentine K (2019). _MOTE:
Measure of the Effect: Package to assist in effect size
calculations and their confidence intervals_. R package version
1.0.2, <URL: http://github.com/doomlab/MOTE>.

stringr : Hadley Wickham (2019). stringr: Simple, Consistent Wrappers for
Common String Operations. R package version 1.4.0.
https://CRAN.R-project.org/package=stringr

gtools : Gregory R. Warnes, Ben Bolker and Thomas Lumley (2018). gtools:
Various R Programming Tools. R package version 3.8.1.
https://CRAN.R-project.org/package=gtools

qdapTools : Rinker, T. W. (2015). qdapTools: Tools to Accompany the qdap
Package. 1.3.2. University at Buffalo. Buffalo, New York.

http://github.com/trinker/qdapTools

ggplot2: H. Wickham. ggplot2: Elegant Graphics for Data Analysis. Springer-Verlag New York, 2016.

tidyverse: Wickham et al., (2019). Welcome to the tidyverse. Journal of Open Source Software, 4(43), 1686, https://doi.org/10.21105/joss.01686

ggpubr : Alboukadel Kassambara (2020). ggpubr: ggplot2 Based Publication Ready Plots. R package version 0.4.0. https://CRAN.R-project.org/package=ggpubr

rstatix : Alboukadel Kassambara (2020). rstatix: Pipe-Friendly Framework for Basic Statistical Tests. R package version 0.6.0. https://CRAN.R-project.org/package=rstatix

SOfun : Ananda Mahto, Jota and Ed Morton (2020). SOfun: Functions From Answers to R Questions on Stack Overflow. http://mrdwab.github.io/SOfun, https://github.com/mrdwab/SOfun.

Hmisc : Frank E Harrell Jr, with contributions from Charles Dupont and many others. (2020). Hmisc: Harrell Miscellaneous. R package version 4.4-1. https://CRAN.R-project.org/package=Hmisc

car : John Fox and Sanford Weisberg (2019). An {R} Companion to Applied Regression, Third Edition. Thousand Oaks CA: Sage. URL: https://socialsciences.mcmaster.ca/jfox/Books/Companion/

broom : David Robinson, Alex Hayes and Simon Couch (2020). broom: Convert Statistical Objects into Tidy Tibbles. R package version 0.7.1. https://CRAN.R-project.org/package=broom

gridExtra : Baptiste Auguie (2017). gridExtra: Miscellaneous Functions for
"Grid" Graphics. R package version 2.3.
https://CRAN.R-project.org/package=gridExtra

knitr : Yihui Xie (2019). knitr: A General-Purpose Package for Dynamic
Report Generation in R. R package version 1.24.

Yihui Xie (2015) Dynamic Documents with R and knitr. 2nd
edition. Chapman and Hall/CRC. ISBN 978-1498716963

Yihui Xie (2014) knitr: A Comprehensive Tool for Reproducible
Research in R. In Victoria Stodden, Friedrich Leisch and Roger
D. Peng, editors, Implementing Reproducible Computational
Research. Chapman and Hall/CRC. ISBN 978-1466561595

corrplot : Taiyun Wei and Viliam Simko (2017). R package "corrplot":
Visualization of a Correlation Matrix (Version 0.84). Available
from https://github.com/taiyun/corrplot

plyr : Hadley Wickham (2011). The Split-Apply-Combine Strategy for Data
Analysis. Journal of Statistical Software, 40(1), 1-29. URL
http://www.jstatsoft.org/v40/i01/.
